# Need for Culturally Competent and Responsive Cancer Education for African Immigrant Families and Youth Living in the United States

**DOI:** 10.2196/53956

**Published:** 2024-03-06

**Authors:** Olufunmilola Abraham, Adeola Agoke, Kazeem Sanuth, Abimbola Fapohunda, Motolani Ogunsanya, Megan Piper, Amy Trentham-Dietz

**Affiliations:** 1 Social and Administrative Sciences Division School of Pharmacy University of Wisconsin-Madison Madison, WI United States; 2 African Cultural Studies University of Wisconsin-Madison Madison, WI United States; 3 National African Language Resource Center Indiana University Bloomington Bloomington, IN United States; 4 Behavioral and Community Health Sciences School of Public Health University of Pittsburgh Pittsburgh, PA United States; 5 College of Pharmacy Health Sciences Center University of Oklahoma Oklahoma City, OK United States; 6 Department of Medicine and Center for Tobacco Research and Intervention School of Medicine and Public Health University of Wisconsin-Madison Madison, WI United States; 7 Population Health Sciences and Carbone Cancer Center School of Medicine and Public Health University of Wisconsin-Madison Madison, WI United States

**Keywords:** African immigrant, youth, adolescent, adolescents, teen, teens, teenager, teenagers, cancer awareness, health disparities, culturally competent education, African, Black, immigrant, cultural, culturally, cancer, oncology, patient education, awareness, prevention, disparity, disparities

## Abstract

Cancer prevalence data for Black Americans is monolithic and fails to consider the diverse cultures and backgrounds within that community. For instance, African immigrants constitute a meaningful proportion of the foreign-born Black immigrants in the United States (42%), but the prevalence of cancer in the African immigrant community itself is unknown. Therefore, without accurate cancer prevalence data, it is impossible to identify trends and other key factors that are needed to support the health of African immigrants and their children. Moreover, it is impossible to understand how the culture and language of subgroups influence their cancer-related health behavior. While research in this area is limited, the existing literature articulates the need for culturally responsive and culturally tailored cancer education for African immigrants and their adolescent children, which is what we advocate for in this viewpoint paper. Existing projects demonstrate the feasibility of culturally responsive programming for adults; however, few projects include or focus on adolescents or children born to African immigrants. To best meet the needs of this understudied community, researchers must use culturally competent interventions alongside familiar, usable media. For adolescents, technology is ubiquitous thus, the creation of a culturally tailored digital intervention has immense potential to improve cancer awareness and prevention for youth and their community. More research is needed to address many of the existing research gaps and develop a rich understanding of the unique experience of cancer among African immigrant families that can be used to inform intervention development. Through this viewpoint, we review the current state of cancer-related research among African immigrant families in the United States. In this paper, we acknowledge the current knowledge gaps and issues surrounding measurement and then discuss the factors relevant to designing an educational intervention targeted at African immigrants and the role of African immigrant youth.

## Introduction

Cancer has a profound impact on the experience of health for many in the United States that only continues to grow. Research has demonstrated the escalating rates of early-onset cancer diagnosis among women and the alarming decreasing rates for men and Black people; most commonly in breast, thyroid, and colorectal cancer [1]. Early diagnosis and prompt treatment of cancer are critical to improved public health. The decreasing rate of early cancer detection and response is introducing a significant health inequity among Black people in the United States. African-born immigrants and their children comprise a meaningful portion of the US population. The paper aims to describe the existing research gaps and experiences of cancer among African immigrant families and highlight the need to design and tailor cancer education for African immigrant families.

There was a surge in the African immigrant population between 1970 and 2015 [2]. This migration pattern has continued, with the African immigrant population growing from 881,000 in 2000 to 2.0 million in 2019, comprising 42% of the US foreign-born Black population. African immigrants have tended to settle in 4 main cities in the United States: Washington DC, New York City, Minneapolis or St Paul, and Atlanta [3]. Prior research has established that most African immigrants come from Western (35.7%) and Eastern Africa (29.6%) [4]: from Nigeria (13.7%), Ethiopia (10.8%), Ghana (7.8%), and Kenya (5.5%) [5]. Therefore, Nigeria and Ethiopia constitute the top birthplaces of African immigrants in the United States.

In 2011, one of the first peer-reviewed papers on African immigrant health was published. It highlighted the growing population of African immigrants in the United States and the need to study their health care needs and practices since chronic diseases, including cancer, were poorly understood [3]. There is a growing research interest in African immigrant health, especially by researchers who are first- and second-generation African immigrants themselves, especially in light of the myriad of factors that impact African immigrants’ health, and that of their families, including the trauma of immigration, conflicting cultural contexts between African family dynamics and those common in the United States, diet and lifestyles, culture, religion, and spirituality. These constructs make up the richness of immigrants’ lives and continue to impact their health behaviors, health care experiences, and decision-making about their health practices after they move to the United States. Therefore, attention must be given to these factors. These factors also influence how African immigrants interact with and navigate the health care system, making it essential to understand how these factors can negatively impact health care system involvement.

The number of health-related areas influenced by immigration only grows as their length of stay in the United States increases [6]. Given the unique health experiences of African immigrants, the need to better understand the cancer-related health of African immigrants is imperative. The United States has begun to address disparities in immigrant health, such as affordances of health care following the implementation of the Affordable Care Act or state-level legislation allowing immigrants (especially the young, pregnant persons, and older people) to be eligible for state Medicare. States that have expanded care for immigrant children have seen reductions in no-insurance rates and rates of families forgoing medical care [7]. However, there is a current dearth of initiatives directly designed for African immigrants. With a deeper understanding of cancer in the African immigrant community, we can create novel, innovative, and culturally specific educational tools to support African immigrant families and improve current and future African immigrant community cancer health.

## Discerning Cancer Prevalence Among African Immigrants

### Overview

Uncovering cancer prevalence and awareness among African immigrants is challenging. Racial and ethnic minority groups are underrepresented in health research overall, contributing to persistent health disparities in the United States [8]. Cancer death rates among Black people continue to increase and so does the risk of developing cancers [9]. In the past few decades, there have been focused efforts to conduct research and draft policies to address health disparities within immigrant communities. However, there is a discernable lack of research on African immigrants’ (and their children’s) health related to cancer prevention and awareness in the United States. The challenge is due to limited resources allocated to minority issues and a lack of effort to distinguish the population as different and unique from other minority populations [10,11].

### Issues With Measurement

Most research on immigrant health in the United States has focused on Latinos and Asian Americans [12-15]. Similarly, most cancer-related research in the United States has used race, and Hispanic or Latino ethnic affinity, regardless of the differing histories of migration, as the basis of categorizing research participants. Therefore, there is limited knowledge about African immigrant health in the United States, especially on cancer awareness, cancer care, and overall health outcomes. Accurate prevalence of cancer among African immigrants is unknown, available literature mostly focuses on databases that have combined data for African-born immigrants and US-born Black people. This makes it difficult to identify African immigrants and to provide accurate evidence of the extent and impact of cancer within their communities [16]. This practice facilitates a monolithic view of people with African heritage; therefore, it discourages granularity of analysis and limits health services’ researchers’ ability to address African immigrant-specific health challenges and examine related research questions.

Some researchers have started to address the overgeneralization of categorizing all African immigrants as “Black” by focusing on their country of origin or time since immigration or assimilation or acculturation [17,18]. Assimilation allows immigrants to integrate into the social, linguistic, and cultural fabric of the host society. However, acculturation experience differs across immigrant groups. The Hispanics, specifically Mexican Americans, constitute an immigrant group in the United States with a robust acculturation. Safran Williams in his classification of diasporas describes Mexican American as “not true diaspora” [19]. This is because of their immigration history with the United States [19]. Further, Spanish is the dominant language of the Mexicans and is also the most popular foreign language in the United States. As a result, acculturation for Mexican immigrants is steady and impacts the strength of research and health interventions for this immigrant group [20-22]. Contrarily, the cultural and linguistic significance of African immigrant identities do not share the same history and recognition both in the United States social milieu and in the US health care system especially those relating to cancer education and research. African immigrants have an existing cultural identification from their homeland and their languages do not have the same recognition as that of Hispanic Americans. Nonetheless, the effort to acculturate among African immigrants accounts for the experiences such as changes in diet, modified language practices, and using the health care services for access to information, treatment, and care. The acculturation process is also layered with the African immigrants’ spirituality and how it influences their reception of health care treatment. Careful attention to the cultural practices of African immigrants and their relevance to health intervention will largely impact the outcomes in cancer awareness and education.

The issue of having a monolithic “Black” category affects the extraction of research data on African immigrants [16]. Some progress is being made in this area. For instance, 1 study promoted awareness and accessibility to screening for chronic diseases among African immigrants living in Georgia [17]. Other research has discussed African immigrants’ health and allostatic load score as it relates to cardiovascular, metabolic, and immune systems [18]. Finally, a scoping review identified additional socio-ecological challenges faced including the lack of culturally competent health care, distrust of the health care system, challenges navigating the US health system, and the burdensome cost of care [16].

## What We Do Know About African Immigrants and Cancer

Accurate prevalence rates of cancer in African immigrants in the United States are lacking. Evidence suggests high cancer prevalence in their countries of origin, especially breast and cervical cancer for women and prostate cancer for men [23-25]. More research is needed to understand the prevalence of cancer within immigrant families and how their immigration may influence cancer prevalence.

The experiences and needs of African immigrants are unique [17,26-32]. Sociocultural factors underlie the experience of cancer in the African immigrant community. The stigma of being diagnosed with cancer, lack of cancer awareness, limited or no screening (especially among African immigrant women), and limited familiarity with prevention strategies and treatment technologies available may be contributing to the high prevalence of cancer [24,30]. These factors lead to late-stage diagnoses because of a lack of access to health care, lower education levels, and cultural and religious beliefs regarding cancer [33,34]. Studies also found that African-born women have limited knowledge and exposure to breast cancer screening information before their arrival in the United States [30,34,35], which can impact their preventative and cancer screening behaviors. Existing research has also explored cancer mortality among adults across different Black ethnic groups—African, African American, and Caribbean—showing some mortality and prevalence differences between these groups [36].

Further, 1 study has found that income, among other factors, plays a significant role in the population’s understanding of colorectal cancer [37]. With a focus on breast and cervical cancer screening, other studies examined the knowledge and perspectives of African immigrants [38,39]. Their findings underscore significant factors impacting the decision to seek preventative screening measures among African immigrants, including fatalism, lack of cancer knowledge, stigma, length of stay in the United States, provider gender, and privacy concerns [40-43]. Another study examined prostate cancer risk experiences among West African men and shed light on the modifiable risk factors implicated in prostate cancer mortality and morbidity [44]. A study of cervical cancer awareness among African immigrant women in Iowa City highlighted factors such as fear, languages spoken, and education as barriers to preventative treatment [45]. Considering the available research and prevalent factors that limit cancer prevention knowledge and behavior it is imperative to develop culturally, and linguistically appropriate cancer education programs aimed at increasing awareness and screening of cancer. In summary, while research has begun to address differences in African immigrant health, the differences are many which will require further study and consensus.

## Lack of Cancer Awareness Among Youth and African Immigrants

In 2008, it was estimated that the 82% of the US population increase between 2005 and 2050 would be attributed to immigrants and their descendants [26]. Despite an increase of African immigrants’ offspring in the United States, little is known about these second-generation individuals born and raised in the United States (with at least 1 foreign-born parent), regarding their health beliefs, perceptions, and practices. This is understandable as little is known about their parents regarding these factors. A study that explored beliefs and lifestyle behaviors relating to healthy living and diet among middle-aged adults in the immigrant population indicated that little is known about the beliefs, perceptions, and practices of diet and exercise among young African immigrants [46]. Young adults of African immigrant descent are part of the future, and attention needs to be paid to their well-being.

It is unknown if children of African immigrants are being educated about cancer by their parents, their communities, their health care providers, or in schools. Cancer is often termed as a taboo subject in most African homes and communities. This is further compounded by other barriers such as access to care, quality of care, communication gaps, lack of education, lack of affordable health care, lack of transportation, socioeconomic status, shame and stigma, and cultural and religious beliefs [47]. Nonetheless, some children of African immigrants become aware of cancer when close family members or friends are diagnosed. With limited cancer awareness and the vulnerability of African immigrants regarding cancer, youth, and their parents must be educated using culturally competent, tailored, and responsive family-oriented cancer education initiatives that build on the strengths of these immigrant cultures as well as address the barriers to cancer prevention behaviors.

Although the limited research reviewed above examines cancer among Black immigrant men and women, there is no substantial body of research that addresses cancer education and awareness among first and second-generation African immigrant adolescents in the United States. A lack of knowledge about youth immigrants and second-generation African immigrants can put this population at a disadvantage as compared to their peers. Cancer awareness among African immigrants and youth studies, including older and younger Somali women, use age as a factor for examining standardized prevalence of cardiovascular disease risk factors among both African immigrants and African Americans [47,48]. Although age is an important factor to consider, this work does not focus on youth. Another study, rather than age, used the year of residence in the United States to examine self-reported health problems among African immigrant adults [49].

While several studies have begun to address cancer research among the African population broadly, the significant paucity of research that focuses on the youth of African immigrant families in the United States leaves a critical gap in cancer awareness and prevalence research. To our knowledge, no studies have sought to examine or address cancer awareness among the youth of African immigrant families, nor interventions for cancer awareness and education. The youth of African immigrant families in the United States constitute an important population that is instrumental in creating awareness about the prevalence of cancer within their community. To access the youth groups of African immigrant descent in the United States, it is expedient to identify cultural and age-relevant educational tools for creating awareness about the prevalence of cancer disease.

## Existing Studies on the Promotion of Cancer Awareness and Education Among African Immigrants

### Overview

There is evidence of studies that promote cancer health education among African immigrants and other minority groups using various culturally tailored approaches and technologies. The success of a community-academic partnership model at community faith-based centers is effective for immigrant women in learning about breast cancer [50]. Moreover, health education programs in community-based settings have indicated strong potential. Further, 2 studies involving interpreters and culturally targeted communication, showed increased breast cancer knowledge and an improvement in screening for breast cancer for immigrant and multicultural women [51,52].

Study findings have demonstrated the importance of culturally tailored educational tools and different approaches to reduce cancer-related disparities. These studies provide strong evidence supporting the use of culturally relevant educational materials, patient navigation programs, peer-to-peer education, education programs, videos, and cofacilitated health promotion forums in promoting preventative and cancer screening behaviors [33,53-62]. Together these projects shed light on some of the few, yet variable opportunities for successful community-engaged research with African immigrant families.

Furthermore, some studies have demonstrated the potential of technology in promoting cancer awareness and education among African immigrants. Mobile devices, tablets, and computers have been used to address common cultural and linguistic barriers to cancer screening. Mobile health initiatives, culturally tailored messaging, language support, mobile apps, short message services, and text messages have all proven effective in impacting cancer screening behaviors [18,63-65]. Some of these initiatives could be adapted into family-based programs where young African immigrants could learn in familiar spaces using ubiquitous and widely acceptable technologies such as serious games.

### Global Health Perspectives and Solutions for Culturally Competent Care Among African Immigrants

Health care approaches for immigrant populations require adaptation and cultural competence to serve diverse communities effectively. Parallel analysis of the US health care models with those of other nations like Canada and Australia offers a framework to evaluate and refine strategies to address health disparities among African immigrants. Canada and Australia have made strides in fostering inclusive health strategies that can inform US health care practices, particularly in providing culturally competent care to African immigrants.

For example, in Canada, health care delivery to immigrant populations acknowledges the necessity of cultural competence. Canada’s universal health care system actively integrates culturally tailored interventions. The Canadian government has pushed for strategies that involve community engagement and representation in health decision-making, enhancing the cultural appropriateness of health care services [66]. Using community health workers who share the same cultural background as immigrants has been a breakthrough, acting as a bridge between health care providers and immigrant communities [67]. These community health workers facilitate communication, understanding, and trust—essential elements in promoting the health and well-being of immigrant populations [68].

Further, Australia’s approach to immigrant health pivots on inclusivity and health equity to deliver services that are respectful of and responsive to diverse patients’ health beliefs, practices, and needs [69]. A notable instance is the Victorian Immigrant and Refugee Women’s Coalition’s efforts, which engage women directly to educate about health issues, including cancer awareness [70]. Australian health policies aim to address the language barriers and the diverse cultural contexts that can influence health care usage and outcomes. In contrast, the United States continues to grapple with creating a standardized approach for culturally competent care throughout its health care system.

While there are pockets of exemplary practices, such as using patient navigators in cancer care to assist patients from minority backgrounds, there is not a universal health care mandate specifically aimed at immigrant health [67]. Instead, the United States relies on a patchwork of local initiatives and federal guidelines, such as those by the Office of Minority Health which established the National Standards for Culturally and Linguistically Appropriate Services in health and health care [71]. In conclusion, both the Canadian and Australian models underscore the importance of cultural competence and systemic support in improving immigrant health outcomes. They demonstrate that effective immigrant health strategies require the integration of culturally informed practices across all stages of health care—from preventive education to treatment. This implies adopting multifaceted approaches that can cater to the unique cultural, linguistic, and religious elements that define African immigrant communities.

## Youth: the Bridge for Culturally Tailored Cancer Education

Given their positionality, first through second-generation African immigrant youth are at a unique nexus from which they can bridge health gaps related to cancer that arise from their heritage and sociocultural contexts. Cultural tailoring acknowledges the broad culture but identifies specific strategies for reaching specific individuals. These groups of individuals have insights into the linguistic and cultural practices of their families as well as those of the society they live in. Due to their positionality, the youth are motivated to embrace language awareness, which emphasizes the interrelatedness of language, culture, and social structures [72]. The interrelatedness of cultural meanings and linguistic signs allows for the tailoring of educational content that addresses distinctive groups. The adolescents of African immigrant families are a product of the diverse linguistic and cultural interactions that occur through transnational migration and globalization.

To engage with youth and form a robust bridge between coexisting sociocultural systems to improve African immigrant community health, research should focus on methods that are familiar and usable for adolescents. A ubiquitous facet of adolescent life is technology. There is increasing interest in serious games (ie, games that serve an educational or developmental purpose aside from pure entertainment) as a learning medium. Although innovative interventions including serious games are becoming popular, they are not traditionally designed and tailored to meet the cultural and health needs of minoritized populations such as African immigrant families. Systemic reviews of serious games indicate limitations that need to be addressed [73-76]. It will be beneficial for health services’ researchers to use a participatory design approach when designing cancer education and intervention tools for African immigrant families. Such a collaborative approach will allow African immigrant families to partner in the co-design of technologies such as serious games and facilitate the creation of a culturally competent and responsive learning medium. Youth from African immigrant families typically have a hybrid of identities which necessitate the use of education technologies such as serious games in ways that speak to their lived experiences and families’ cultural heritage and realities. Therefore, there is a need to tailor educational resources using technology platforms that would engage the linguistic and sociocultural realities of the African immigrant population. Interventions to improve cancer outcomes in African immigrants, especially among youth, are necessary.

Youth and community members from other minority populations in the Northwest Arctic region of Alaska participated in community-based participatory action research honoring indigenous ways, creating a Sharing Circle used to understand community priorities and develop culturally relevant cancer education that could be incorporated into school curriculum. It is an opportunity for youth involvement in culturally relevant health promotion efforts to address health disparities in cancer [77].

## Culturally Tailored Education for African Immigrant Youth

### Overview

Developing educational resources for African immigrant youth brings into focus the question of curricular content and pedagogical approaches that fit this group. The connection of educational content with cultural identities is espoused in the framework of culturally relevant pedagogy (CRP) [78]. CRP encompasses multiple concepts related to students’ academic achievements and social inequalities, but its central tenet is the interconnection of theories and cultures in manners that will “empower students intellectually, socially, emotionally, and politically by using cultural referents to impart knowledge, skills, and attitudes [79].” African immigrants and people of historically marginalized cultures are unique and deserving of an educational approach that is aligned with their needs. It offers liberatory education which inspires the learners to become social commentators, advocates, and critical consumers of knowledge while empowering control over one’s health. The use of such an approach will be beneficial in disseminating and promoting cancer education in the community.

The pedagogical approaches to achieving culturally tailored education may derive from CRP and adopt effective strategies that will merge critical consciousness and cultural connections in the learning content. CRP proposes three components that must be integrated to achieve learning: (1) a focus on youth learning and academic success, (2) developing youth’s cultural competence to assist them in developing positive ethnic and social identities, and (3) supporting youth’s critical consciousness or their ability to recognize and critique societal inequalities.

Researchers have described examples of targeted and tailored strategies, techniques, and procedures for successful intervention with a variety of populations [80]. These researchers identified linguistic, community-engaged, and sociocultural strategies as important to reaching a particular community. Building on this knowledge, we identify four approaches that a cancer education intervention that the youth of African immigrant heritage can draw on, namely: (1) linguistic and cultural markers, (2) belief system and religious affiliation, (3) hybrid nationality, and (4) age-related learning preferences. With a deeper understanding of how these factors, concerning cancer health, shape the identities, beliefs, and behaviors of African immigrant youth in the United States, we may be able to create culturally competent educational tools for cancer awareness and prevention.

### Linguistic and Cultural Markers

African immigrants, having come from different countries with diverse colonial histories, have distinct languages. The native languages of African immigrants play an important part in their identity. The youth of African immigrants assimilate the language and cultures of the host society while leveraging their cultural and linguistic heritage for optimum survival, a process that yields linguistic and cultural hybridity.

The complexity that underlies the African immigrants’ linguistic and cultural identities in the United States should inform approaches to developing culturally competent education for youth and their families to improve overall health outcomes. It is expedient to target cancer-awareness information by incorporating aspects of the home languages of African immigrants—especially Western and Eastern Africa [5]. For example, the Swahili language would be accessible to immigrant families of East African origin, and Pidgin English for families with West African heritage. Appropriate learning mediums for cancer awareness for African immigrant youth should intersect with the linguistic and cultural practices of the African immigrant population.

### Belief System and Religious Affiliation

In a 2021 report, the Pew Research Center stated that African immigrants in the United States are more religious than other Black Americans, even though Black Americans are more religious than Americans of other races [81]. Further broken down into specific practices, the report noted that African immigrants value attending religious services weekly, more than other Black Americans: “around half of the African immigrants living in the United States (54%) say they attend religious services at least weekly, compared with about 3-in-10 United States-born (32%) and Caribbean-born (30%) Black adults.”

Similar to language, culture, and national consciousness, the belief systems and religiosity of African immigrants will have a major imprint on their young children. Health information tailored specifically to religiosity will not only be responsive to African immigrants’ cultural perspectives, but it may also improve engagement with pedagogical materials. Moreover, studies are scarce on the intersection of African immigrants’ religious practices and responses to health care education about cancer, thereby illustrating another gap in research that may ultimately improve the approach to cancer education among distinctive ethnic and racial groups. Additionally, there is a shortage of research on the religious practices of African immigrants, highlighting another research gap that could ultimately enhance approaches to cancer education among distinct ethnic and racial groups.

### Hybrid Nationality and Afropolitanism

African immigrants in the United States, have diverse origins from one of the 54 nations of Africa, many of which are multiethnic. These diverse ethnic identities house unique cultural and linguistic features within and outside the individual nation’s borders. While African immigrants actively engage with the dominant Western traditions of the society they reside in, they also maintain their cultural customs. As a result, youth from African immigrant families often exhibit hybrid language use, blending the host language with elements of African culture, including specific exclamation and colloquial forms rooted in African cultural beliefs. This linguistic and cultural hybridity is significant in addressing the existing gap in cancer awareness research among African immigrant families and fosters a sense of community within the African immigrant population in the United States.

The concept of Afropolitanism defines Africans as an integral part of the global community rather than separate from it. This concept refers to the empowerment associated with a blended, polyethnic, and cosmopolitan identity [80]. Afropolitanism iterates Africans’ awareness of their origins and the consciousness of the cultural ambiguities that occur because of their integration into the host society. This understanding impacts African immigrants’ response to cancer education and approaches to accessing health care for cancer treatment. Their cultural and spiritual beliefs are still very much prominent in their perspective on cancer disease. This consciousness could, however, be tapped into for possible changes and adaptations among this immigrant group. The summary of the African immigrants’ complex experience is iterated in the term, “Afropolitan.” Afropolitan describes an individual whose identities are deeply rooted in their diverse, transcultural experiences, reflecting youth linguistic and cultural practices within African immigrant families [68,69]. African immigrants’ hybrid language and cultural identities necessitate the development of health educational tools and technologies that integrate African cultural perspectives and engage these youth in learning and retaining health information in a culturally responsive manner.

### Age-Related Learning Preferences

Consideration for age-appropriateness in technology is not unique to African immigrant youth; however, the connection of this factor to digital literacy, access, and equity makes it critical to examine further and worthy of discussion. A report by the Migration Policy Institute on immigrant learning with digital technology has identified uneven access to digital resources for youth aged between 15 and 17 years who are either immigrants themselves or have at least one immigrant parent [82]. Research suggests that factors like work, language, and familial influence affect how immigrant youth use technology for learning [83]. Given the versatility of the adolescent age group with technology, they have increased access to vital information on health issues and diseases that are prevalent within their community. More important is their access to their heritage culture as well as the culture of their residing society. As a result, youth play a vital role as intermediaries, connecting with their families to promote cancer awareness within their communities.

## Further Research Needed

### Overview

A robust foundation of data and associated knowledge surrounding cancer in the communities of African immigrants is needed to truly understand the impact of cancer on this group and appropriate approaches to intervention to reduce cancer risk and improve cancer treatment. Several priorities are highlighted throughout this paper and an overview is presented in Textbox 1.

**Summary of key main areas for future research.**

**Priorities for future cancer prevention and control research focused on African immigrant populations**

- Disaggregate study populations according to country or region of origin to improve cultural tailoring and knowledge.
- Develop family-oriented educational initiatives including programs for children.
- Use community-engaged approaches including partnerships with faith-based organizations.
- Leverage emerging technology for recruiting study participants and delivering educational messages while accounting for barriers to access.
- Align cancer awareness information with language and cultural markers specific to the population.
- Consider the global African community and hybrid African and American cultural practices.
- Incorporate relevant religious and spiritual beliefs and practices to enhance cancer education effectiveness.
- Consider youth and adolescents as intermediaries for increasing cancer awareness among family members.
- Explore the potential for interagency collaboration (Centers for Disease Control and Prevention, Centers for Medicare & Medicaid Services, Health Resources and Services Administration, and National Institutes of Health) to address cancer-related health challenges for African immigrant families.

To achieve the goal of increasing cancer awareness among African immigrant families, 1 strategy involves creating a culturally tailored serious game. Serious games offer opportunities to build upon the research base of effective approaches to reduce the cancer burden by focusing on youth and leveraging technology. Research is crucial that examines the language use of youth from African immigrants in the United States. Previous research has already categorized most African immigrants living in the United States into Western (35.71%) and Eastern Africa (29.61%) groups, which could serve as a basis for examining youth cancer awareness within each group [5]. Open-ended ethnographic interviews could be used to identify the nuanced cultural and linguistic practices of the youth of African immigrant families. The heterogeneity of Africa’s cultural identities could result in a new monolithic idea of Black subgroups in the United States, the importance of beginning this inquiry cannot be delayed. Detailed demographic questionnaires and open response forms can allow for flexibility in how studies aggregate and allow for new divisions and aggregations of African immigrants. However, it is noted that immigration by African countries is unequal with many African immigrants arriving from Western and Eastern African countries [5].

Additionally, recruitment strategies are particularly important in the success of this line of research and will need to be evaluated. As immigrant populations are “Hard-to Reach,” using innovative ways to reach a target population is also important [84]. During the COVID-19 pandemic, online recruitment using Facebook (Meta), Instagram (Meta Platforms), and WhatsApp (WhatsApp LLC) was an effective recruitment strategy especially because it built on existing communication and information-sharing norms within the African immigrant community. Further research should use and evaluate multiple recruitment streams.

Findings from such research endeavors will have a meaningful impact on the strategies for developing culturally tailored educational content such as a serious game, to create awareness about cancer among African immigrant families in the United States. A culturally adapted serious game has immense potential to be instrumental in improving awareness and cancer prevention strategies in African immigrant families.

### Conclusion

The importance of culturally tailored cancer education for African immigrants is underscored by uncertainty. Issues surrounding the measurement of cancer prevalence in African immigrant populations exacerbate the uncertainty of how cancer affects the African immigrant population in the United States. The existing, yet limited research on the topic suggests that African immigrants, especially adolescents, have unique experiences that lie at the nexus of their traditional culture and the complex novelty of the US health care system for immigrants. Research demonstrates the impact of cultural beliefs (such as fatalism and stigmatization of cancer diagnoses among African cultures) and lack of knowledge about cancer and cancer screening compounds to affect access to screening and care for African immigrants. Further research specifically targeting African immigrants and their youth can not only disentangle the unique struggles of African immigrants but also allow for the tailoring of education to provide maximal impact to vulnerable populations.

While recognizing our lack of knowledge and the uncertainty around the experience of cancer in the United States for African immigrants and advocating that increased research is the needed foundation for alleviating health disparities, more difficult work is ahead. It is integral for health scientists, health care providers, African culture scholars, and communities of African immigrants to come together for sustained research activity. These transdisciplinary associations will aid in the collection of data specific to African immigrants, but also the community engagement needed to co-design a culturally sensitive educational intervention. This will be no small task and require the dedicated work of many experts alongside and within the African immigrant community to forge long-term relationships that can facilitate recruitment, retention, and meaningful knowledge generation for the African immigrant community in the context of cancer experience.

## Abbreviations

CRP: culturally relevant pedagogy
